# 4-(Hydroxymethyl)catechol Extracted From Fungi in Marine Sponges Attenuates Rheumatoid Arthritis by Inhibiting PI3K/Akt/NF-κB Signaling

**DOI:** 10.3389/fphar.2018.00726

**Published:** 2018-07-20

**Authors:** Jong Y. Lee, Geum J. Kim, Jin K. Choi, Young-Ae Choi, Na-Hee Jeong, Pil-Hoon Park, Hyukjae Choi, Sang-Hyun Kim

**Affiliations:** ^1^Department of Pharmacology, School of Medicine, CMRI, Kyungpook National University, Daegu, South Korea; ^2^College of Pharmacy, Yeungnam University, Gyeongsan, South Korea; ^3^Molecular Immunology Section, Laboratory of Immunology, National Eye Institute, National Institutes of Health, Bethesda, MD, United States

**Keywords:** collagen-induced arthritis, synovial fibroblasts, 4-(hydroxymethyl)catechol, *Pestalotiopsis*, matrix metalloproteinase, inflammatory cytokine

## Abstract

Rheumatoid arthritis (RA) is a progressive autoimmune disease specific to synovial joints; it causes joint damage and other systemic abnormalities, thereby leading to physical disability and early mortality. Marine sponge-derived fungi, *Pestalotiopsis* sp., secrete immunosuppressive compounds in the culture broth. In the present study, we isolated 4-(hydroxymethyl)catechol (4-HMC) from these fungal species, and evaluated its anti-RA effects using a murine collagen-induced arthritis model and tumor necrosis factor-α-stimulated human RA synovial fibroblasts. Oral 4-HMC administration decreased the clinical arthritis score, paw thickness, histologic and radiologic changes, and serum IgG1 and IgG2a levels. It prevented the proliferation of helper T (Th) 1/Th17 CD4^+^ lymphocytes isolated from inguinal lymph nodes, thereby reducing inflammatory cytokine production in CIA mice. It decreased the expression of inflammatory mediators, including cytokines and matrix metalloproteinases (MMPs), both *in vitro* and *in vivo*. We observed that 4-HMC suppresses Th immune responses and MMP expression to inhibit inflammatory cytokine production in human RA synovial fibroblasts by modulating the PI3K/Akt/NF-κB pathway. These results verify the anti-RA potential of 4-HMC.

## Introduction

Rheumatoid arthritis (RA) is characterized by articular inflammation, synovial joint damage, and bone destruction over time (McInnes et al., 2016). It can cause chronic pain and severe disability, thereby increasing mortality. Among its various causes, lymphocytes are known to be essential for the pathogenesis of RA. Phagocytic cells of the innate immune system, including macrophages and dendritic cells, are implicated in RA progression (Cuda et al., 2016). These cells activate immune cells and promote their influx into the synovial tissue. Synovial fibroblasts in synovium produce cytokine, chemokines, and matrix metalloproteinases (MMPs) (Haleagrahara et al., 2018). MMPs drive cartilage erosion in RA (Noss et al., 2011). Particularly, MMP-1 and MMP-3 play a pivotal role in tissue destruction. They are produced by synovial fibroblasts and monocytes/macrophages in the synovium (Varani et al., 2011). The PI3K/Akt/NF-κB signaling axis is known to be important for RA pathogenesis (Jia et al., 2015). PI3K is largely associated with cell growth, migration, proliferation, and differentiation. It activates Akt, which then stimulates NF-κB to secrete pro-inflammatory cytokines, such as TNF-α, IL-1β, and IL-6 (Pringle et al., 2012; Tas et al., 2016).

In general, the treatment of RA focuses on reducing articular inflammation. Disease-modifying anti-rheumatic drugs and glucocorticoids have exhibited a significant treatment potential; they were introduced in 1998. Numerous studies have demonstrated the efficacy of methotrexate and dexamethasone (Dexa) (Boers et al., 1997; Fleischmann, 2015). Despite their advantages, their use is limited by two main factors. First, they are not universally effective to RA patients; many patients do not adequately respond to therapy with these drugs (Weinblatt et al., 2013). Second, chronic glucocorticoid treatment drastically reduces intestinal calcium absorption and induces a significant degree of bone loss (Gennari, 1993; Canalis and Delany, 2002). The combined use of TNF-α inhibitors and DMARDs is recently applied in clinical use, which shows positive therapeutic effects, such as alleviation of the symptoms, prevention of bone destruction, and improvement in physical function (Ma and Xu, 2013). Although the early treatment of TNF-α inhibitors and DMARDs for RA is known to be effective, the majority of patients who are not treated in the early stage suffers from several side effects, such as joint pain, joint swelling, and fatigue (Dogra and Khullar, 2013; Monaco et al., 2015).

Since the ocean is an area that remains underutilized in terms of its biological resource, it has a great potential for biomedical applications (Cragg and Newman, 2013). Marine sponges have been reported to be a good source of bioactive extracts/compounds possessing anti-inflammatory, anti-oxidant, anti-viral, and anti-bacterial activities (Mencarelli et al., 2014; Lin et al., 2015; Di et al., 2017). *Pestaloptiosis* sp. are endophytic fungi that are common in tropical plants (Yang et al., 2012). This genus has interested many researchers because it is abundant in natural bioactive products. A phthalide derivative isolated from *P. photiniae* is reported to induce apoptosis in HeLa cells (Chen and Yang, 2013). Polyketide derivatives isolated from *P. clavispora* show anti-cancer activities (Perez Hemphill et al., 2016). Caryophyllene sesquiterpenoids isolated from *Pestalotiopsis* sp. inhibit macrophage-produced nitric oxide (NO) (Liu et al., 2016). Due to the known anti-inflammatory effect of marine sponge and effectiveness of *Pestalotiopsis* sp. on the NO production which is an important mediator of inflammation, we isolated 4-(hydroxymethyl)catechol (4-HMC) from *Pestalotiopsis* sp., and investigated its anti-inflammatory actions on cytokine production in RA.

## Materials and Methods

### General Experimental Procedures

Silica gel (230–400 mesh, Merck KGaA, Darmstadt, Germany) column chromatography was performed with dichloromethane and methanol as eluents. HPLC was performed with a Gilson system (Gilson Inc., Middleton, WI, United States) using a Shim-pack ODS (21.5 × 250 mm, Shimadzu, Kyoto, Japan) column. NMR spectra (^1^H and ^13^C) were recorded on a Bruker Avance DPX 250 Spectrometer (Bruker, Billerica, MA, United States) using CD_3_OD. LR-ESI-MS was performed on an Agilent 6120 series LC-MS (Agilent Technologies, Santa Clara, CA, United States).

### Isolation and Fermentation of the Fungal Strain

The marine fungi (*Pestalotiopsis* sp.) were isolated from marine sponges collected off the coast of Jeju Island, South Korea in 2012. Based on a 99% 18S rRNA sequence similarity, the fungi were identified as *Pestalotiopsis* sp. and cultured in 30 L of sea water containing glucose (1 g/L), yeast extract (0.1 g/L), and peptone (0.5 g/L) at room temperature for 18 days.

### Extraction and Isolation

Cultured mycelia were filtered out from the broth, which was then extracted twice with ethyl acetate. After evaporation under vacuum, the crude extract (2 g) was subjected to silica gel vacuum chromatography eluted with dichloromethane and methanol to obtain seven fractions. Fraction 6 (324.8 mg) was purified by HPLC [Shim-pack ODS (21.5 × 250 mm) column, 6 mL/min, UV 210 nm, ACN: H_2_O = 5:95 → 20:80 (60 min)] to yield the final compound (39 mg, Rt = 31.8 min), 4-(hydroxymethyl)catechol (1): pale brown oil; LR-ESI-MS, *m/z* 163.1 [M + Na]^+^ (C_7_H_8_O_3_Na); ^1^H-NMR (CD_3_OD, 250 MHz) δ 6.76 (H-3, 1H, *d, J* = 2.8), 6.62 (H-6, 1H, *d, J* = 8.6), 6.54 (H-5, 1H, *J* = 8.6, 2.8), and 4.59 (H-7, 2H, *s*); ^13^C-NMR (CD_3_OD, 63 MHz) δ 151.1 (C-1), 148.9 (C-2), 129.5 (C-4), 116.7 (C-5), 115.8 (C-3), 115.5 (C-6), and 61.1 (C-7) (Supplementary Figures S1–S3 and Supplementary Table S1).

### Animals

The 6-week-old male DBA/1J mice (total *n* = 30) were purchased from Orient Bio (Seoul, South Korea), and housed in a laminar airflow room, maintained at 22 ± 2°C, 55 ± 5% relative humidity, and a 12-h light:dark cycle throughout the study. All mice were provided *ad libitum* access to standard rodent chow and filtered water during the study. Food and water consumption, as well as body weight gain, were recorded. Care and treatment of the mice were conducted in accordance with the guidelines established by the Public Health Service Policy on the Humane Care and Use of Laboratory Animals. The study protocol was approved by the Institutional Animal Care and Use Committee of Kyungpook National University.

### Establishment and Assessment of the Arthritis Model

A murine collagen-induced arthritis (CIA) model was established as previously described with minor modifications (Nam et al., 2013). Complete Freund’s adjuvant (4 mg/mL, Chondrex, Redmond, WA, United States) and bovine type-2 collagen (2 mg/mL, Chondrex) were emulsified in a 1:1 ratio using a tissue homogenizer at 4°C for 20 min. After visually confirming that the emulsification is complete, the emulsion was filled in an immunization syringe; all mice were subcutaneously injected in the tail over 2 min with 100 μg of the emulsion (primary immunization). After 21 days, the mice were injected with a 1:1 emulsion of bovine type-2 collagen and incomplete Freund’s adjuvant (secondary immunization). Three groups (*n* = 5) of mice were orally administered 4-HMC at 2, 10, and 50 mg/kg 19 times between days 28 and 53 after the initial immunization; dexamethasone (Dexa) (1 mg/kg) was administered as the standard treatment (*n* = 5). 4-HMC and Dexa were dissolved in dimethyl sulfoxide and diluted in phosphate-buffered saline (PBS) before use. Unimmunized vehicle control mice (*n* = 5) and CIA disease control mice (*n* = 5) were orally administered PBS alone. The degree of arthritis was determined on a scale of 0–4 (0, no arthritis; 1, one inflamed digit; 2, two inflamed digits; 3, more than two inflamed digits and an inflamed footpad; and 4, all digits were inflamed). The cumulative score of four paws of each mouse was used as the clinical arthritis score (maximum score, 16) to represent overall disease severity and progression. The incidence of arthritic paws was defined as the occurrence of inflamed paws with a clinical arthritis score of 2 or more. Paw thickness was measured using a dial thickness gauge (Mitutoyo Co., Tokyo, Japan). Hind limb joints were assessed radiographically on day 55 and were evaluated on a scale of 0–3 (1, soft tissue swelling only; 2, soft tissue swelling and early joint erosion; 3, severe bone erosion or significant osteophyte formation; maximum score, 6).

On day 56, the mice were euthanized by carbon dioxide and whole blood from the celiac artery was collected. After leaving the blood samples undisturbed at room temperature and allowing them to clot, they were centrifuged at 2000 ×*g* for 15 min at 4°C, and the supernatant serum samples were isolated. Further, the paws were dissected and processed for histopathological analysis. Total serum IgG1 and IgG2a levels were measured using ELISA kits (BD Biosciences, Oxford, United Kingdom) according to the manufacturers’ instructions.

### Micro-Computed Tomography (CT)

The right femurs of mice were excised, fixed in 4% paraformaldehyde for 16 h, and scanned using a SkyScan 1272 high-resolution micro-CT system (Bruker, Kontich, Belgium) with a source voltage of 60 kV, current of 166 μA, and resolution of 14 μm. The data were analyzed using the CTAn software (Bruker).

### Micro-Positron Emission Tomography (PET)

Micro-PET imaging was performed with an Inveon PET/CT Scanner (Siemens Medical Solutions, Knoxville, TN, United States). The mice were anesthetized under 1–2% isoflurane and [^18^F] FDG (18.5 MBq in 150 μL of saline) was injected via the tail vein. The anesthetized mice were then placed prone on the scanner bed and imaged at 1 h after [^18^F] FDG injection; the imaging duration was 20 min. Images were reconstructed using a two-dimensional ordered-subset expectation maximum algorithm, and [^18^F] FDG uptake in the wrist and ankle joints were quantified at a coronal plane by ellipsoid region of interest analysis. Activity accumulation was expressed as a percentage of the decay-corrected injected dose per gram using the AMIDE software. No corrections were necessary for attenuation or scatter. The PET images were visualized using the Inveon Research Workplace software.

### Flow Cytometry

At the end of the experiment, inguinal lymph nodes were collected from each mouse and ground using 70-μm nylon cell strainers (Falcon, Bedford, MA, United States) to isolate single cells. Subsequently, the cells were stained with a mouse Th1/Th17 phenotyping kit (BD Biosciences) according to the manufacturer’s instructions. Markers for Th1/Th17 were CD4 PerCP-Cy5.5-FITC-conjugated Th1 (IFN-γ), and CD4 PerCP-Cy5.5-PE-conjugated Th17 (IL-17A), respectively. Cells were stimulated for 4 h with phorbol myristate acetate and BD Cytofix^TM^ Fixation Buffer. Cell stimulation was terminated by fixing in 4% formyl saline. Fixed cells were stained in 0.1% BD Perm/Wash^TM^ Buffer for 30 min and finally analyzed on a FACSCalibur (BD Biosciences). The forward and side scatter gating method, a commonly used method in flow cytometer, was utilized for this analysis.

### Cell Culture and Cell Viability Analysis

The RA synovial fibroblasts were isolated by enzymatic dispersion of synovial tissues from RA patients as previously described (Nam et al., 2013; Choi et al., 2015). Synovial tissue samples were obtained from a 44-year-old female patient with RA at the time of joint surgery and used in this study. To confirm our results, we additionally obtained synovial tissue samples from 59- to 68-year-old female patients with RA. The isolated cells were cultured as a monolayer. Written informed consent was obtained from all patients in accordance with the Declaration of Helsinki and the protocols were approved by the Institutional Ethics Committee (IRB: 2052-040903). RA synovial fibroblasts were maintained in Dulbecco’s modified Eagle’s medium supplemented with 10% fetal bovine serum and antibiotics (100 U/mL, penicillin G; and 100 μg/mL, streptomycin) at 37°C in 5% CO_2_. The fibroblasts were used after 3–8 passages for further experiments. Cell viability was determined using the MTT assay (Han et al., 2014). After 24 h of treatment with different concentrations of 4-HMC, MTT (5 mg/mL) was added to each well, and the cells were incubated for 2 h. Isopropanol was added to dissolve the formazan crystals, and the absorbance of each sample was expressed as a percentage relative to control.

### Histological Analysis

The hind paws were fixed with 10% formaldehyde for 2 days and decalcified in 10% EDTA for 30 days at 4°C. The decalcified paws were dehydrated in a gradient ethanol series (70–100%), washed twice with xylene, and embedded in paraffin. Subsequently, 5-μm sections were stained with H&E. A blinded observer scored all the sections from each mouse on a scale of 0–3 (0, normal; 1, infiltration of inflammatory cells; 2, synovial hyperplasia and pannus formation; and 3, bone erosion and destruction; maximum score, 3).

### Cytokine Expression Analysis Using qPCR

The qPCR was carried out to measure cytokine expression using the Thermal Cycler Dice TP850 real-time system (Takarabio, Shiga, Japan) according to the manufacturer’s protocol. At the end of the animal experiment, the entire left hind paw was excised after removal of the skin, and total RNA was isolated. Human RA synovial fibroblasts (2 × 10^5^ cells/well in 24-well plates), were pretreated with 4-HMC or Dexa for 1 h, followed by stimulation with TNF-α (10 ng/mL) for 12 h. Total cellular RNA was isolated from the cells using RNAiso Plus (Takarabio), and cDNA was synthesized using RT Premix (iNtRON Biotech, Sungnam, Korea). Reverse transcription was conducted at 45°C (60 min) and 95°C (5 min). Briefly, 2 μL of cDNA (100 ng), 1 μL each of sense and anti-sense primer solutions (0.4 μM), 12.5 μL of SYBR Premix Ex Taq (Takarabio), and 9.5 μL of dH_2_O were mixed to obtain the final reaction mixture (25 μL). The list of primers is shown in Supplementary Table S2. mRNA expression was normalized and quantified using the TP850 software supplied by the manufacturer.

### Nuclear Protein Extraction

The RA synovial fibroblasts (2 × 10^6^ cells/well in a 6-well plate) were pretreated with 4-HMC or Dexa for 1 h, followed by stimulation with TNF-α (10 ng/mL) for 30 min. After stimulation, the cells were washed in 1 mL of ice-cold PBS, centrifuged at 1,200 ×*g* for 5 min, and resuspended in 400 μL of ice-cold hypotonic buffer (10 mM HEPES/KOH, 2 mM MgCl_2_, 0.1 mM EDTA, 10 mM KCl, 1 mM DTT, and 0.5 mM PMSF, pH 7.9). Further, the cells were left on ice for 10 min, vortexed, and centrifuged at 15,000 ×*g* for 30 s. After washing, the pelleted nuclei were resuspended in 50 μL of ice-cold saline buffer (50 mM HEPES/KOH, 50 mM KCl, 300 mM NaCl, 0.1 mM EDTA, 10 % glycerol, 1 mM DTT, and 0.5 mM PMSF, pH 7.9) and kept on ice for 20 min. Subsequently, the nuclei were vortexed and centrifuged at 15,000 ×*g* for 5 min at 4°C, and the supernatant was isolated.

### Western Blot Analysis

Samples for Western blot analysis were prepared as described previously (Choi et al., 2016). Briefly, cells (2 × 10^6^ cells/well in a 6-well plate) were stimulated with TNF-α (10 ng/mL) to induce the production of various signaling molecules; the cells were incubated with TNF-α for 15 min (PI3K and Akt), 30 min (IKKα/β, N-NF-κB, and IκBα), or 18 h (MMP-1 and MMP-3). They were then rinsed twice with ice-cold PBS, and total cell lysates were collected in 200 μL of lysis buffer. The lysates were centrifuged for 20 min at 4°C and the supernatants were collected. The proteins were electrophoresed using 8–12% SDS–PAGE and transferred onto nitrocellulose membranes. Immunodetection was performed using the Super Signal West Pico Chemiluminescent Substrate (Thermo Scientific, Waltham, MA, United States).

### Statistical Analysis

Statistical analyses were performed using Prism 5 (GraphPad Software, San Diego, CA, United States) and treatment effects were analyzed using one-way ANOVA followed by Dunnett’s test; *p* < 0.05 was considered statistically significant. For mean clinical arthritic and histological scores, the nonparametric Mann–Whitney *U*-test was used to identify significant differences between the experimental groups and the disease-control group.

## Results

### Isolation of 4-HMC and Structure Identification

The 4-HMC was isolated from the ethyl acetate extract of the culture broth of the marine fungi, *Pestalotiopsis* sp., as a pale brown powder; LR-ESI-MS revealed the [M + Na]^+^ ion at *m/z* 163.1. ^1^H NMR ABX spectra revealed proton signals at δ_H_ 6.76 (H-3, 1H, *d, J* = 2.8 Hz), 6.62 (H-6, 1H, *d, J* = 8.6 Hz), and 6.54 (H-5, 1H, *J* = 8.6, 2.8 Hz); an oxygenated methylene proton signal was observed at δ_H_ 4.59 (H-7, 2H, *s*). ^13^C NMR spectra exhibited two phenolic carbon signals (δ_C_ 151.1 and 148.9), four aromatic carbon signals (δ_C_ 129.5, 116.7, 115.8, and 115.5), and an oxygenated methylene carbon signal (δ_C_ 61.1) (Supplementary Figures S2, S3 and Supplementary Table S1). After comparing these spectroscopic data with those reported in the literature (Du et al., 2011), the structure of the isolated compound was determined.

### Therapeutic Effects of 4-HMC on RA Progression

**FIGURE 1:**
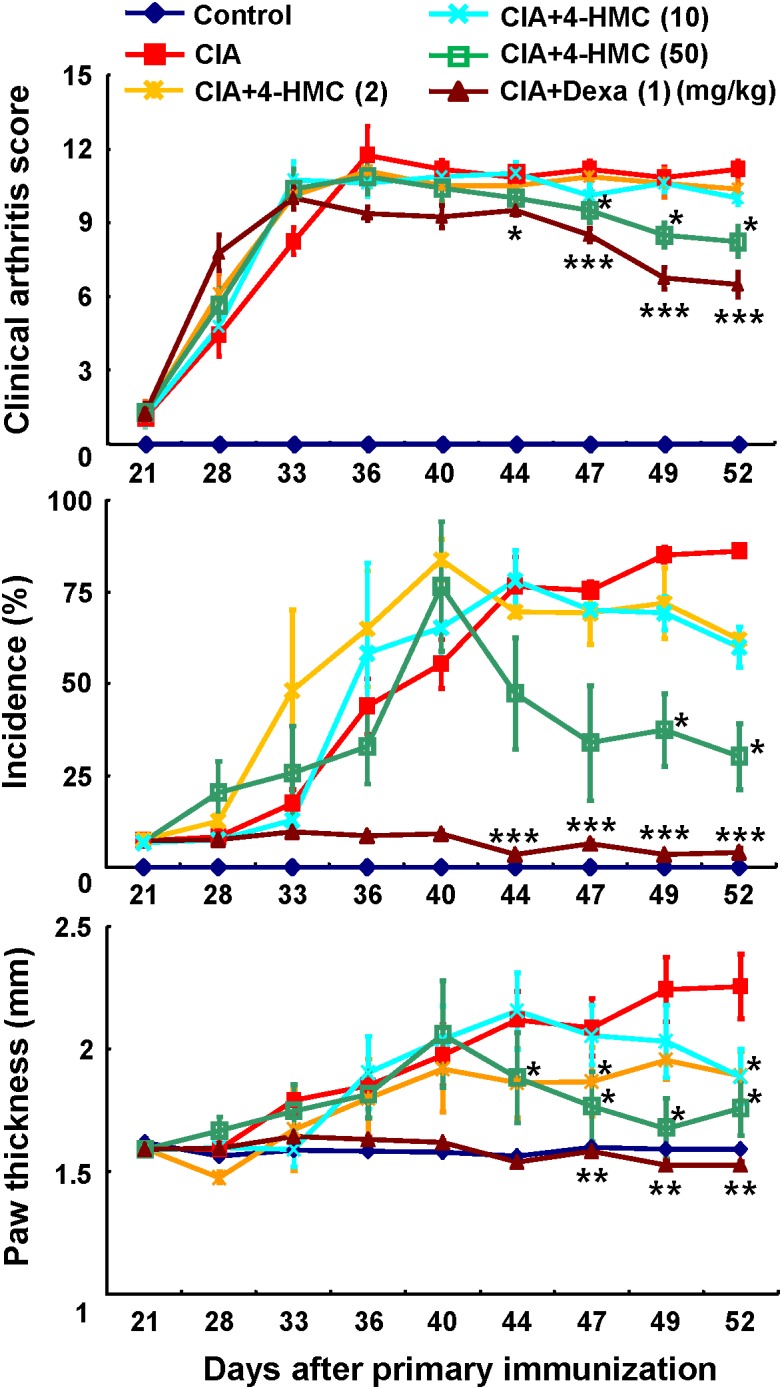
Analysis of the progression of rheumatoid arthritis (RA) using a murine collagen-induced arthritis (CIA) model. Mice with CIA were orally administered 4-HMC or Dexa from day 28 after the primary immunization. RA severity was monitored based on the clinical arthritis score, the incidence of arthritic paws, and paw thickness, which was measured using a dial thickness gauge. The data are presented as the mean ± SD of five determinations. ^∗∗∗^*p* < 0.001; ^∗∗^*p* < 0.01; ^∗^*p* < 0.05, significantly different from untreated CIA mice. 4-HMC, 4-(hydroxymethyl)catechol; Dexa, dexamethasone.

**FIGURE 2:**
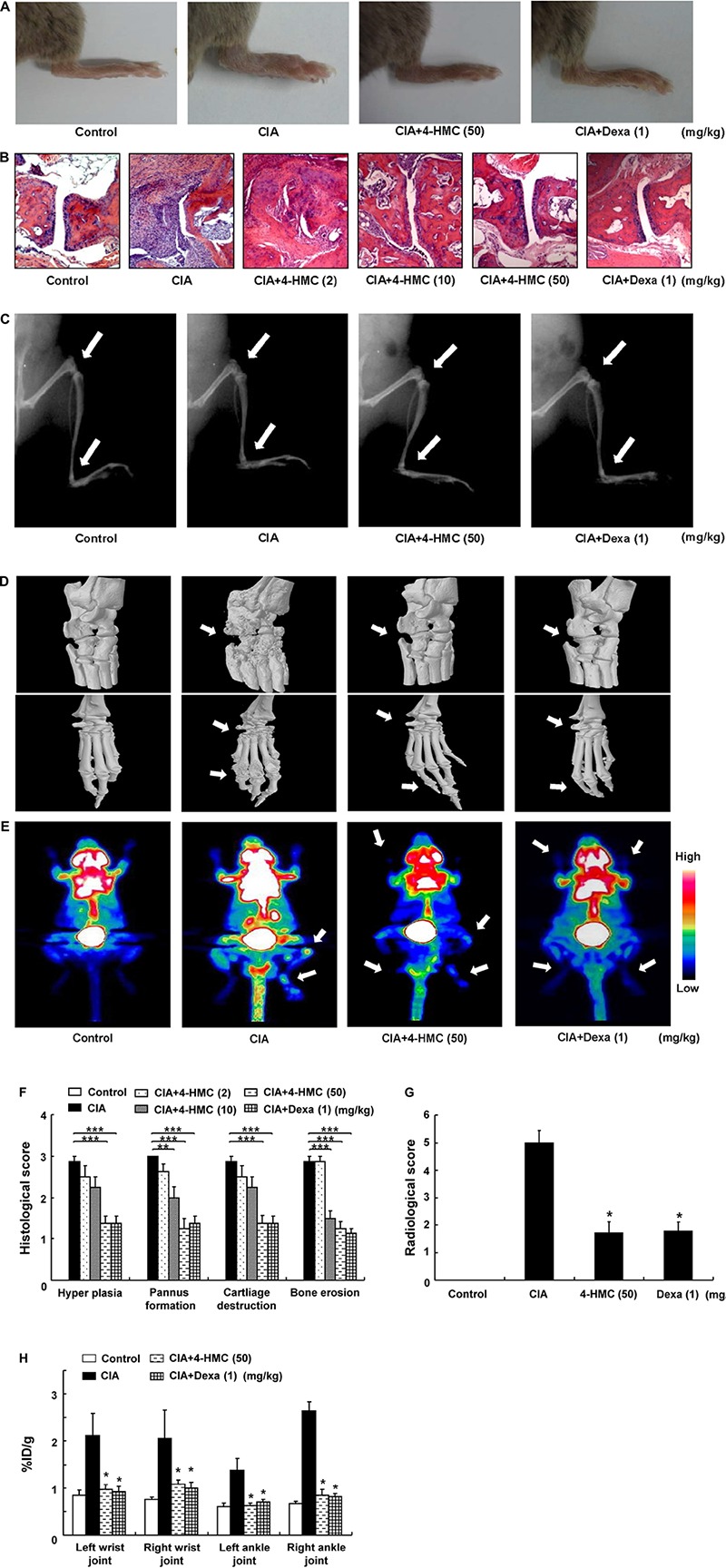
Histologic, radiologic, micro-computed tomography (CT), and [^18^F] FDG micro-positron emission tomography (PET) changes in collagen-induced arthritis (CIA) mice. **(A)** Representative clinical images of the hind paws. **(B)** Representative photomicrographs of paw sections stained with H&E (original magnification × 100). **(C)** Representative radiographic images of the hind paws. **(D)** Representative micro-CT images of the ankle joints. **(E)** Representative maximum intensity projection PET images; [^18^F] FDG accumulation at the site of inflammation was observed by glucose metabolism. **(F)** Histological scores were determined after observation of the H&E sections. **(G)** Radiological scores were determined as described in the section “Materials and Methods.” **(H)** Estimation of rheumatoid arthritis severity based on micro-PET imaging. The data are presented as the mean ± SD of five determinations. ^∗∗∗^*p* < 0.001; ^∗∗^*p* < 0.01; ^∗^*p* < 0.05, significantly different from untreated CIA mice. 4-HMC, 4-(hydroxymethyl)catechol; Dexa, dexamethasone.

The 4-HMC was orally administered to CIA mice at 2, 10, or 50 mg/kg daily from day 28 to day 53 after immunization. The detailed experimental schedule is shown in Supplementary Figure S4. The assessment of the clinical symptoms of arthritis was continued until 52 days after the secondary immunization. Dexa was used as the standard treatment drug. The mean body weight and food/water consumption were not significantly altered by 4-HMC treatment (Supplementary Table S3) indicating no adverse effect of 4-HMC. After 44 days post immunization, 4-HMC (50 mg/kg)-treated CIA mice showed decreased clinical arthritis scores, disease incidence, and paw thickness (**Figure 1**). Histological and radiological scores of severe RA inflammation, characterized by edema, pannus formation, synovial hyperplasia, cartilage degeneration, and bone erosion, were observed in untreated CIA mice (**Figures 2A–C,F–G**). However, 4-HMC-treated mice exhibited less severe RA. Further, the therapeutic effect of 4-HMC was confirmed by observing gross bone changes, such as erosions and joint deformity, using micro-CT and micro-PET imaging. Although bone surfaces were significantly degraded in untreated CIA mice, those in 4-HMC-treated mice showed defects only in two digits. No bone degradation was found in Dexa-treated mice (**Figure 2D**). [^18^F] FDG micro-PET is a recently developed *in vivo* imaging tool for screening anti-inflammatory or anti-RA drugs. It allows the visualization of the metabolic activity in inflamed cells (Haavisto et al., 2016). Therefore, we analyzed the accumulation of [^18^F] FDG in the ankle and knee joints of CIA mice. 4-HMC-treated CIA mice showed significantly lesser uptake of [^18^F] FDG in their ankle and knee joints than untreated CIA mice (**Figures 2E,H**). This result indicates that 4-HMC attenuates inflammation in the joints.

### Inhibitory Effects of 4-HMC on the Serum IgG Levels in CIA Mice

**FIGURE 3:**
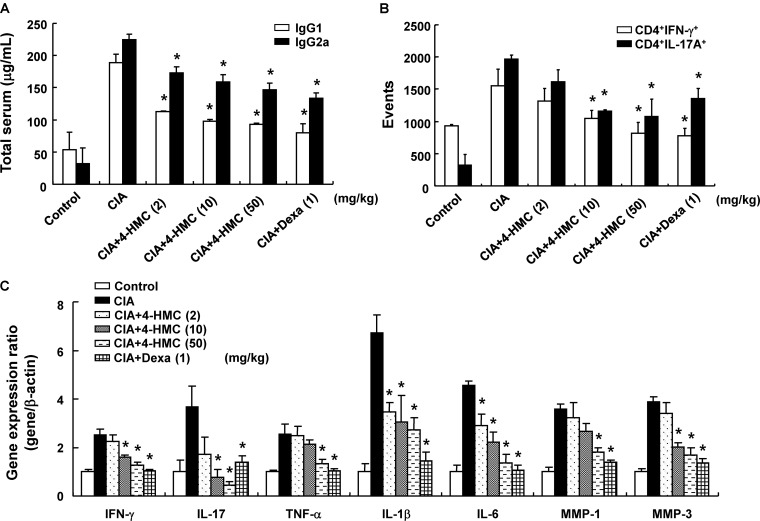
Serum IgG levels, phenotypes of T cells in the lymph nodes, and cytokine expression in the joints of collagen-induced arthritis (CIA) mice. **(A)** Total serum IgG levels were measured by ELISA. **(B)** Inguinal lymph nodes were collected from each mouse, and the numbers of CD4^+^IFN-γ^+^ and CD4^+^IL-17A^+^ isolated single cells were detected using FACSCalibur flow cytometry. **(C)** The joints were excised, total RNA was isolated, and qPCR gene expression analysis was performed. The data are presented as the mean ± SD of five determinations. ^∗^*p* < 0.05, significantly different from untreated CIA mice. 4-HMC, 4-(hydroxymethyl)catechol; Dexa, dexamethasone.

The IgGs are major players of the humoral immune system; they are associated with the promotion of inflammation and RA (Schwartz et al., 2016). Specifically, IgG2a production is linked to Th1 responses, whereas IgG1 production is linked to Th2 responses. CIA is triggered by Th1-mediated autoimmune responses. Patients with early phase RA exhibit elevated Th2 responses in the synovial fluid, whereas these responses are absent in patients with late phase RA. To verify the effects of 4-HMC on these humoral immune responses in the CIA model, serum samples were obtained from each group of mice on day 56 after the primary immunization. Total serum IgG1 and IgG2a levels were significantly higher in untreated CIA mice than in 4-HMC-treated CIA mice (**Figure 3A**). These results suggest that 4-HMC hinders both Th1 and Th2 immune responses in the acute stage of CIA inflammation.

### Inhibitory Effects of 4-HMC on the Proliferation of Th Cells and the Expression of Inflammatory Cytokines and MMPs in CIA Mice

CD4^+^ T cells are known to be closely associated with the pathogenesis of RA. IFN-γ-producing Th1 cells and IL-17-producing Th17 cells are known to cause joint destruction by promoting synovial inflammation and osteoclast formation (Annunziato et al., 2009). Th1 and Th17 cells induce arthritis by regulating immune responses in mice and humans (Damsker et al., 2010; Park et al., 2014). Mice with CIA exhibit increased levels of IFN-γ and IL-17 expressed by Th1 and Th17 cells in the lymph nodes (Xuzhu et al., 2012). Thus, lymphocytes were isolated from CIA mice 56 days after the primary immunization to verify whether 4-HMC inhibits the proliferation of Th cells to suppress CIA severity. 4-HMC-treated CIA mice showed lower numbers of IFN-γ^+^- and IL-17^+^-CD4^+^ T cells than untreated CIA mice (**Figure 3B**).

Pro-inflammatory cytokines promote the secretion of MMPs, which are early inducers of RA (Smolen et al., 2012). MMPs promote cartilage and bone damage in RA (Yoshioka et al., 2013). Therefore, we evaluated the effect of 4-HMC on the expression of inflammatory cytokines and MMPs in the joint tissues and the serum. Untreated CIA mice exhibited elevated levels of pro-inflammatory cytokines (TNF-α, IL-1β, and IL-6), Th1 (IFN-γ), Th17 (IL-17), and MMPs (MMP-1 and MMP-3); however, treatment with 4-HMC reduced these levels in the joint tissues (**Figure 3C**) and the serum (Supplementary Figure S5).

### Inhibitory Effects of 4-HMC on the Activation of TNF-α-Stimulated RA Synovial Fibroblasts

**FIGURE 4:**
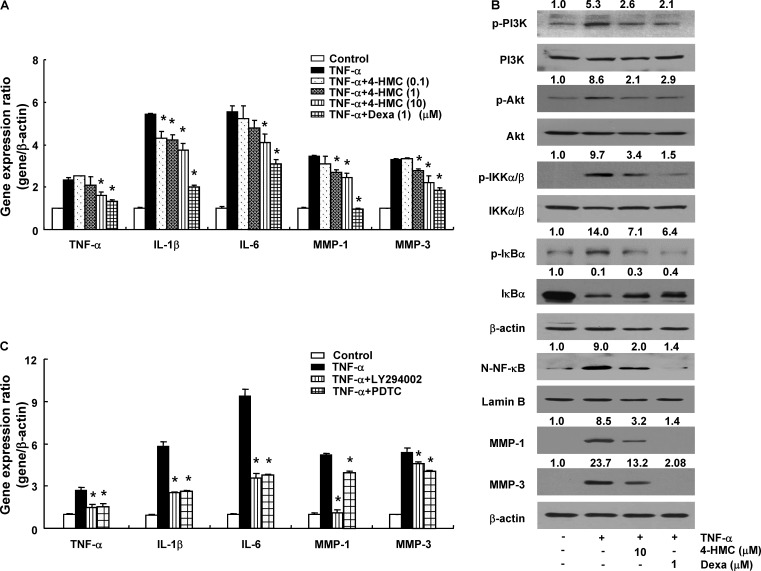
Gene expression and protein synthesis in TNF-α-stimulated rheumatoid arthritis (RA) synovial fibroblasts. **(A)** Cells were pretreated with 4-HMC or Dexa for 1 h before stimulation with TNF-α (10 ng/mL) for 12 h. **(B)** Cells were pretreated with 4-HMC or Dexa for 1 h before stimulation with TNF-α (10 ng/mL) for 15 min (PI3K and Akt), 30 min (IKK α/β, IκBα, and NF-κB), or 18 h (MMP-1 and MMP-3). The data are representative of three independent experiments. The band intensity was digitized and normalized by the relative ratio. N-NF-κB; nuclear NF-κB. **(C)** Cells were pretreated with specific inhibitors of PI3K (LY294002, 20 μM) and NF-κB (PDTC, 20 μM) for 1 h before stimulation with TNF-α (10 ng/mL) for 12 h. The expression of cytokines and MMPs was determined by qPCR. The data are presented as the mean ± SD of three determinations. ^∗^*p* < 0.05, significantly different from TNF-α-stimulated untreated cells. 4-HMC, 4-(hydroxymethyl)catechol; Dexa, dexamethasone.

The TNF-α promotes the secretion of pro-inflammatory cytokines, chemokines, and MMPs (MMP-1 and MMP-3) from synovial fibroblasts (Burrage et al., 2006). 4-HMC did not show cytotoxicity within 24 h up to a concentration of 100 μM in RA synovial fibroblasts (Supplementary Figure S6). The cells were treated with 4-HMC for 1 h and stimulated with TNF-α for 12 h. A significant increase in the expression of pro-inflammatory cytokines (TNF-α, IL-1β, and IL-6) was observed after TNF-α stimulation, whereas pretreatment with Dexa and 4-HMC decreased these levels (**Figure 4A**). To confirm the suppressive effect of 4-HMC, we isolated synovial fibroblasts from two other donors and measured the expression of pro-inflammatory cytokines, chemokines, and MMPs; we found similar suppressive effects (Supplementary Figure S7).

The PI3K/Akt/NF-κB-dependent signaling pathway in RA synovial fibroblasts regulates the expression of pro-inflammatory cytokines (Kumar et al., 2001). Therefore, we pretreated RA synovial fibroblasts with 4-HMC for 1 h and stimulated them with TNF-α for different durations; the cells were stimulated with TNF-α for 15 min (PI3K and Akt), 30 min (IKKα/β, N-NF-κB, and IκBα), or 18 h (MMP-1 and MMP-3). TNF-α stimulation induced PI3K, Akt, and IKKα/β phosphorylation, IκBα degradation, and p65 NF-κB nuclear translocation (**Figure 4B**); however, these effects were suppressed by pretreatment with 4-HMC. In addition, it reduced the TNF-α-stimulated expression and protein levels of MMP-1 and MMP-3 (**Figures 4A,B**). Further, we pretreated RA synovial fibroblasts with specific inhibitors of PI3K (LY294002, 20 μM) and NF-κB (PDTC, 20 μM) before TNF-α stimulation. We observed that both the inhibitors conspicuously decreased the expression of TNF-α, IL-1β, IL-6, MMP-1, and MMP-3 (**Figure 4C**). This experiment was also confirmed with cells from two other donors (Supplementary Figure S8). These results indicate that 4-HMC inhibits the TNF-α-induced expression of inflammatory mediators by regulating the PI3K/Akt/NF-κB pathway.

## Discussion

*Pestalotiopsis* sp. are fungi producing numerous bioactive secondary metabolites that exhibit anti-fungal (ambuic acid) (Li et al., 2001), cytotoxic (torreyanic acid) (Lee et al., 1996), and immunosuppressive activities (pestalotiopsins A and B) (Pulici et al., 1996). In the present study, the compound isolated from halophilic *Pestalotiopsis* sp. was identified as 4-HMC by comparing its spectroscopic data with those reported (Du et al., 2011). This compound has been isolated from virgin olive oils (Saitta et al., 2008) and maple syrup (Zhang et al., 2014). In addition, 4-HMC isolated from an endophytic mangrove fungus (*Aspergillus clavatonanicus*) was reported to be cytotoxic to HeLa cells with an IC_50_ value of 4 μg/mL (Du et al., 2011). In this study, we demonstrated its anti-RA effects *in vivo* and *in vitro*.

We observed that 4-HMC reduces CIA severity as observed by the clinical arthritis score, arthritis indices, paw swelling, and histological changes, including hyperplasia, pannus formation, cartilage destruction, and bone erosion in joints. Radiological analysis showed that it suppresses the progression of cartilage destruction and bone erosion in joints, despite the onset of typical CIA symptoms. Loss of bone volume and density, observed through the use of semi-automated micro-CT imaging analysis, indicated RA progression in untreated mice. 4-HMC suppressed the inflammation of the wrist and ankle joints, observed by micro-PET imaging, which accurately indicates RA severity (dos Anjos and da Mota, 2014; Chaudhari et al., 2016). These findings support that 4-HMC is a promising anti-RA agent.

Serum IgG1 and IgG2a levels significantly influence the development of CIA (Cho et al., 2007). RA is caused by auto antibodies with citrullinated proteins and IgG (a rheumatoid factor) (Andersson et al., 2016). In our model, 4-HMC-treated CIA mice showed lower serum levels of IgG1 and IgG2a than untreated CIA mice by controlling both Th1 and Th2 immune responses. CD4^+^ T cells regulate the immune system. Antigen presentation by dendritic cells activates Th cells of different lineages with distinct biological characteristics (Zhu et al., 2010). Th1 and Th17 cells play an important role not only in the pathogenesis of RA, but also in the human immune system by modulating inflammation and autoimmune responses. (Xuzhu et al., 2012). RA is typically caused due to inflammation triggered by Th1 and Th17 responses (Hu et al., 2014). 4-HMC decreased the proliferation of Th1 and Th17 cells isolated from lymph nodes. Consequently, we could assume that it inhibits RA by regulating T cell responses.

Activated synovial fibroblasts express cytokines, such as TNF-α, IL-1β, IL-6, and IL-17, thereby causing bone destruction (Kumar et al., 2016). We found that 4-HMC suppresses the production of inflammatory cytokines and bone destruction in CIA mice. These inflammatory cytokines promote the synthesis of MMPs, which are matrix-degrading enzymes, responsible for joint destruction through cartilage or bone degradation as well as angiogenesis promotion in RA (Wang et al., 2015). In the case of RA patients, MMPs contribute to cartilage destruction (Otero and Goldring, 2007). Among the various MMPs, MMP-1 and MMP-3 in the joints degrade the extracellular matrix and cause joint damage (Green et al., 2003). We found that 4-HMC inhibits the expression of MMP-1 and MMP-3 to prevent joint damage in CIA mice.

Synovial fibroblasts are crucial for the pathogenesis of RA. Their activation leads to the secretion of numerous pro-inflammatory mediators, resulting in tissue destruction (Shu et al., 2015). TNF-α-induced RA synovial fibroblasts have been used to identify the significance of inflammatory cytokines and MMPs (Yoshioka et al., 2013). Patients with RA exhibit high levels of TNF-α and IL-1β in all joints as well as increased serum levels of MMP-3 (Ribbens et al., 2002; Kay and Calabrese, 2004). Inflammatory cytokines, such as TNF-α and IL-1β, degrade the extracellular matrix by inducing the production of MMPs (Dodge and Poole, 1989). Especially, activated MMP-3 leads to proteoglycan loss. In addition, it stimulates the activation of proMMP-1, which accelerates collagen degradation (Burrage et al., 2006). Thus, it is worthwhile to investigate signal transduction pathways and molecular mechanisms to control MMP expression for discovering novel anti-RA agents that prevent joint damage. We found that 4-HMC inhibited the TNF-α-induced expression of pro-inflammatory cytokines (TNF-α, IL-1β, and IL-6) and MMPs in synovial fibroblasts, verifying the therapeutic potential of 4-HMC for RA treatment.

NF-κB is a major regulator of inflammation in RA. It accelerates the progression of RA by increasing tissue damage, immune cell proliferation, and MMP expression (Shu et al., 2015). Several cytokines, including TNF-α, activate the PI3K/Akt/NF-κB pathway in RA synovial membranes (Tian et al., 2013). Further, Akt level in the synovial tissue is significantly higher in RA than in osteoarthritis, suggesting that RA-mediated inflammation is responsible for the increased Akt level (Tas et al., 2016). Thus, the PI3K/Akt/NF-κB axis influences the production of inflammatory cytokines and synovial cell proliferation, as well as RA progression. Although the specific mechanisms underlying the anti-RA effects of 4-HMC remain elusive, we demonstrate that it acts by regulating PI3K/Akt/NF-κB signaling in synovial fibroblasts.

Commonly used drugs for RA are not free from side effects. For instance, anti-TNF inhibitors cause serious side effects, including cancer, tuberculosis, or pneumonia. In addition, adverse effects of prolonged use of steroids such as Dexa have been well known. The current study focused on 4-HMC as a safe alternative to current anti-inflammatory drugs. Given that 4-HMC is a natural product, it is expected to have less side effects than chemically synthesized drugs (Lahlou, 2013; Nema et al., 2013). However, further studies are needed to clarifying the safety of 4-HMC and therapeutic effects of 4-HMC in other inflammatory diseases.

## Conclusion

This study verified the anti-RA effects of 4-HMC by using both *in vivo* and *in vitro* models of RA. It suppresses the expression of cytokines and MMPs by reducing PI3K/Akt/NF-κB signaling in RA synovial fibroblasts. Thus, our study suggests that 4-HMC is a potential therapeutic candidate for the treatment of RA. In addition, it may be developed as an effective drug for treating various other autoimmune diseases because it is a natural anti-inflammatory drug.

## Author Contributions

HC and S-HK conducted the study. JL, GK, and P-HP designed the detailed experiments, performed the study, and collected and analyzed the data. JC, Y-AC, and N-HJ took part in the cell experiments in this study. All the authors commented the study and approved the final manuscript.

## Conflict of Interest Statement

The authors declare that the research was conducted in the absence of any commercial or financial relationships that could be construed as a potential conflict of interest.

## Abbreviations

4-HMC: 4-(hydroxymethyl)catechol
Akt: v-Akt murine thymoma viral oncogene
CIA: collagen-induced arthritis
CT: computed tomography
MMP: matrix metalloproteinases
NF-κB: nuclear factor-κB
PET: positron emission tomography
PI3K: phosphoinositide 3-kinase
RA: rheumatoid arthritis
