# Supplementary material for: 4-(Hydroxymethyl)catechol Extracted From Fungi in Marine Sponges Attenuates Rheumatoid Arthritis by Inhibiting PI3K/Akt/NF-κB Signaling
Source: Front Pharmacol. 2018 Jul 20;9:726. doi: 10.3389/fphar.2018.00726 (PMC6062625; doi:10.3389/fphar.2018.00726)
Supplement: Supplementary file 1 [file Presentation_1.pdf]

Supplementary Materials

4-(Hydroxymethyl)catechol Extracted from Fungi in Marine Sponges Attenuates Rheumatoid Arthritis by Inhibiting PI3K/Akt/NF-κB Signaling

Jong Yeong Lee, Geum Jin Kim, Jin Kyeong Choi, Young-Ae Choi, Na-Hee Jeong, Hyukjae Choi*, Sang-Hyun Kim*

* **Correspondence**: Sang-Hyun Kim: shkim72@knu.ac.kr and Hyukjae Choi: h5choi@yu.ac.kr

1. Supplementary material and method

Determination of cytokines and MMP1/3

Serum samples were collected at the end of experiments. The levels of mouse serum TNF-α, IL-1β, and IL-6 were quantified by a 20-plex mouse cytokine assays (luminex platform, Invitrogen). MMP-1 was determined using an assay kit (LSBio), and MMP-3 was measured by ELISA kit (R&D Systems) according to the manufacturer's instructions.

Specific inhibitors

LY294002 and pyrrolidine dithiocarbamate (PDTC) were purchased from Calbiochem (La Jolla, CA). RA synovial fibroblasts were pre-treated with LY294002 (20 μM) and PDTC (20 μM) for 1 h, followed by stimulation with TNF-α (10 ng/mL) for 12 h.

2. Legend for supplementary figures

Fig. S1. Chemical structure of 4-(hydroxymethyl)catechol.

Fig. S2. ^{1}H NMR spectrum (250 MHz, CD_3OD) of 4-(hydroxymethyl)catechol.

Fig. S3. ^{13}C NMR spectrum (63 MHz, CD_3OD) of 4-(hydroxymethyl)catechol.

Fig. S4. Experimental design for the induction of collagen-induced arthritis (CIA).

Mice were initially immunized by subcutaneous tail vein injection of 100 µg of bovine type-II collagen emulsified with complete Freund's adjuvant (1:1). A second injection of 100 µg of bovine type-II collagen emulsified with incomplete Freund's adjuvant (1:1) was administered subcutaneously in the tail 21 days later. CIA mice were orally administered 4-HMC or Dexa from day 28 to day 53 after the primary immunization. 4-HMC, 4-(hydroxymethyl)catechol; Dexa, dexamethasone.

Fig. S5. Serum levels of cytokines and matrix metalloproteinases (MMPs).

Blood samples from vehicle-control mice, untreated collagen-induced arthritis (CIA) mice, CIA mice treated with 4-HMC (50 mg/kg), and CIA mice treated with Dexa (1 mg/kg) were collected from the celiac artery on day 56. The data are presented as the mean ± SD of five determinations. $*p < 0.05$, significantly different from untreated CIA mice. 4-HMC, 4-(hydroxymethyl)catechol; Dexa, dexamethasone.

Fig. S6. Effects of 4-HMC on the viability of rheumatoid arthritis synovial fibroblasts.

Cell viability within 24 h was determined by the MTT assay. The data are presented as the mean ± SD of five determinations. *$p < 0.05$, significantly different from control. 4-HMC, 4-(hydroxymethyl)catechol.

Fig. S7. Gene expression in TNF-α-induced rheumatoid arthritis synovial fibroblasts isolated from two other donors was analyzed by qPCR.

The data are presented as the mean ± SD. *$p < 0.05$, significantly different from untreated TNF-α-stimulated cells. 4-HMC, 4-(hydroxymethyl)catechol; Dexa, dexamethasone.

Fig. S8. Effects of specific inhibitors on TNF-α-stimulated rheumatoid arthritis synovial fibroblasts from two other donors.

Cells were pretreated with specific inhibitors of PI3K (LY294002, 20 μM) and NF-κB (PDTC, 20 μM) for 1 h before stimulation with TNF-α (10 ng/mL) for 12 h. Cytokine expression was determined by qPCR. The data are presented as the mean ± SD. *$p < 0.05$ significantly different from untreated TNF-α-stimulated cells.

Table S1. ^{1}H (250 MHz, CD_3OD) and ^{13}C NMR (63 MHz, CD_3OD) data of 4-HMC.

Position	δ_C	δ_H (multiplicity, J in Hz)
1	151.1, C	
2	148.9, C	
3	115.8, CH	6.76 (d, 2.7)
4	129.5, C	
5	116.7, CH	6.54 (dd, 2.8, 8.6)
6	115.5, CH	6.62 (d, 8.5)
7	61.1, CH_2	4.59 (s)

Table S2. List of primers for qPCR experiments.

	Gene	Forward primer (5'-3')	Reverse primer (5'-3')
Human	TNF-α	CCT ACC AGA CCA AGG TCA AC	AGG GGG TAA TAA AGG GAT TG
	IL-1β	GGA TAT GGA GCA ACA AGT GG	ATG TAC CAG TTG GGG AAC TG
	IL-6	AAA GAG GCA CTG GCA GAA AA	ATC TGA GGT GCC CAT GCT AC
	MMP-1	TGG ACC TGG AGG AAA TCT TG	AGT TCA TGA GCT GCA ACA CG
	MMP-3	TTC CTT GGA TTG GAG GTG AC	TGC CAG GAA AGG TTC TGA AG
	β-actin	GGA CTT CGA GCA AGA GAT GG	AGC ACT GTG TTG GCG TAC AG
Mouse	IFN-γ	TCA AGT GGC ATA GAT GTG GAA GAA	TGG CTC TGC AGG ATT TTC ATG
	IL-17	TCC CTC TGT CAT CTG GGA AG	CTC GAC CCT GAA AGT GAA GG
	TNF-α	GGC AGG TCT ACT TTG GAG TCA TTG C	ACA TTC GAG GCT CCA GTG AAT TCG G
	IL-1β	ATA ACC TGC TGG TGT GTG AC	AGG TGC TGA TGT ACC AGT TG
	IL-6	CCG GAG AGG AGA CTT CAC AG	GGA AAT TGG GGT AGG AAG GA
	MMP-1	CCT TCC TTT GCT GTT GCT TC	AGC CCA AAT AAC TGC TGC AT
	MMP-3	AAG TTC CTC GGG TTG GAG AT	TTT CAA TGG CAG AAT CCA CA
	β-actin	TAG ACT TCG AGC AGG AGA TG	TTG ATC TTC ATG GTG CTA GG

Table S3. Effects of 4-HMC on mean body weight, water and food consumption in male DBA/1J mice.

Treatment (mg/kg)	Body weight (mg)	Water consumption (ml)	Food consumption (g)
Control	23.31 ± 1.26	6.21 ± 0.34	4.31 ± 0.25
CIA	21.22 ± 1.53	6.13 ± 0.88	3.81 ± 0.53
CIA+4-HMC (2)	21.42 ± 1.48	6.05 ± 0.53	3.88 ± 0.42
CIA+4-HMC (10)	21.47 ± 1.51	6.12 ± 0.47	3.91 ± 0.48
CIA+4-HMC (50)	23.23 ± 1.36	6.19 ± 0.31	4.27 ± 0.30
CIA+Dexa (1)	23.43 ± 1.23	6.23 ± 0.33	4.29 ± 0.33

The data are presented as the mean ± SD of five determinations.

4-HMC, 4-(hydroxymethyl)catechol; Dexa, dexamethasone.

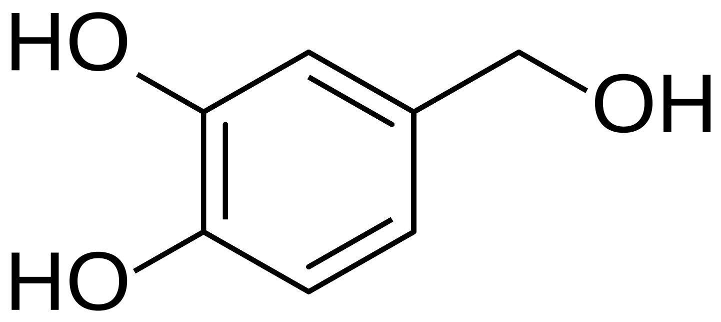

Supplementary Figure S1

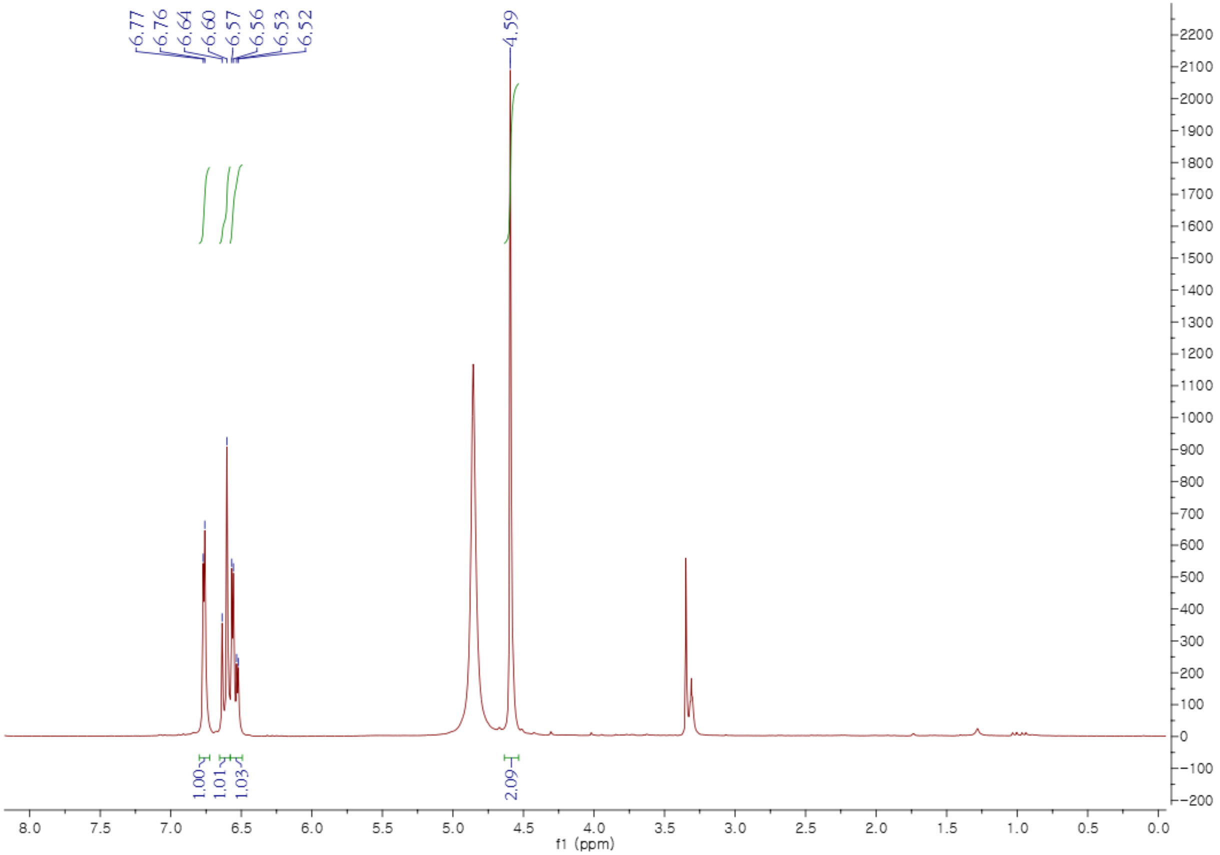

Supplementary Figure S2

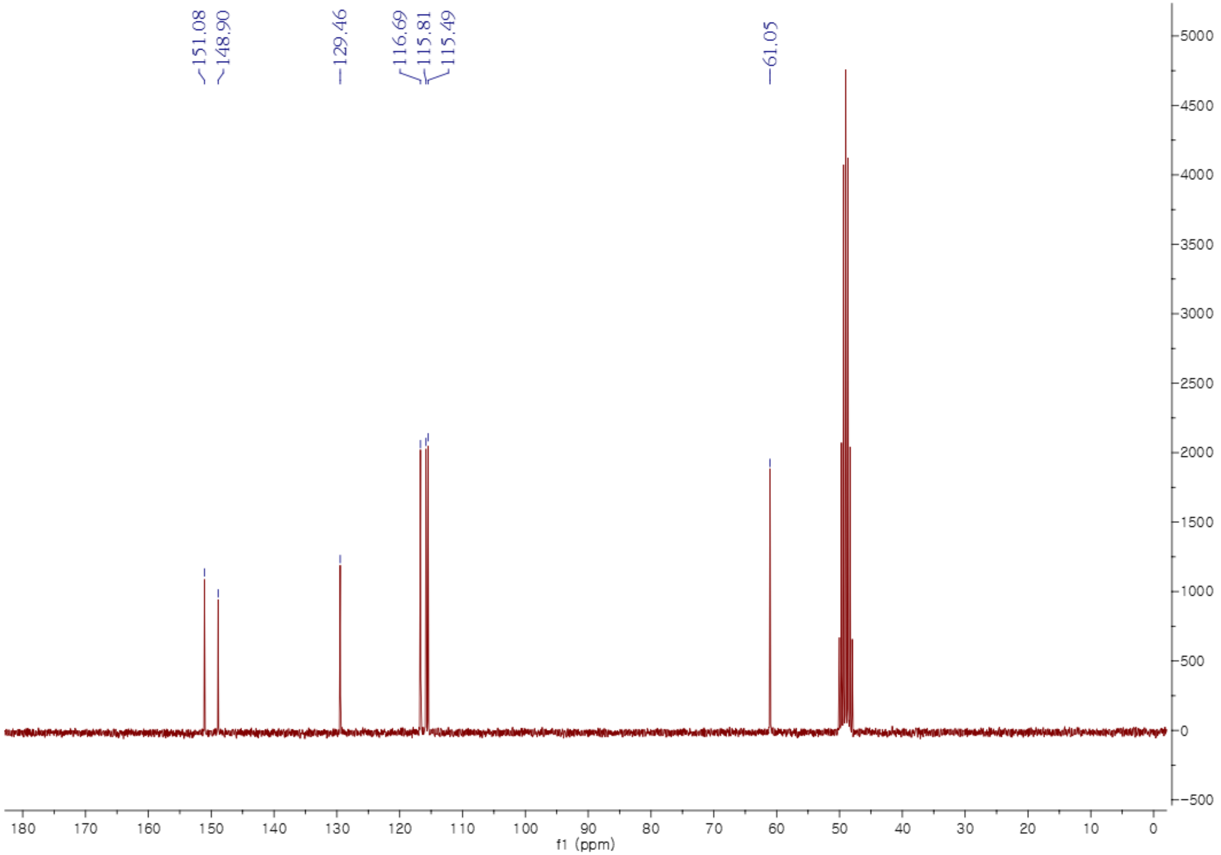

Supplementary Figure S3

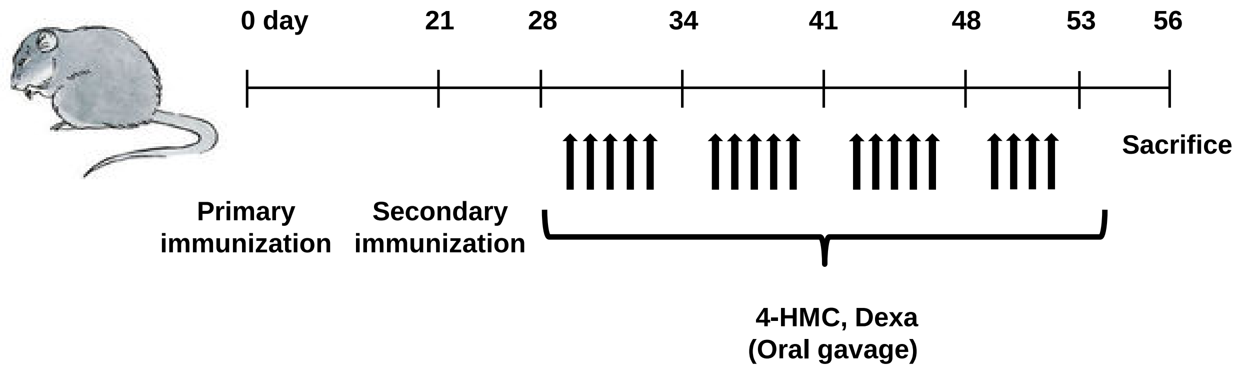

Supplementary Figure S4

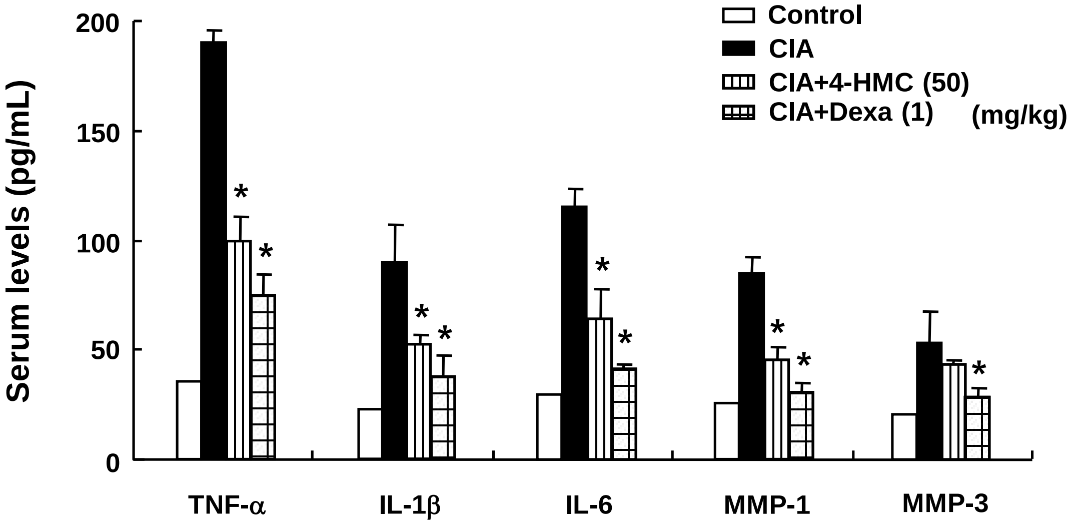

Supplementary Figure S5

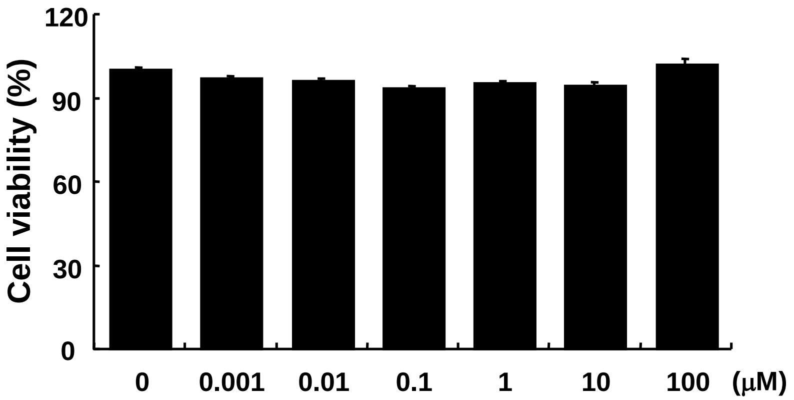

Supplementary Figure S6

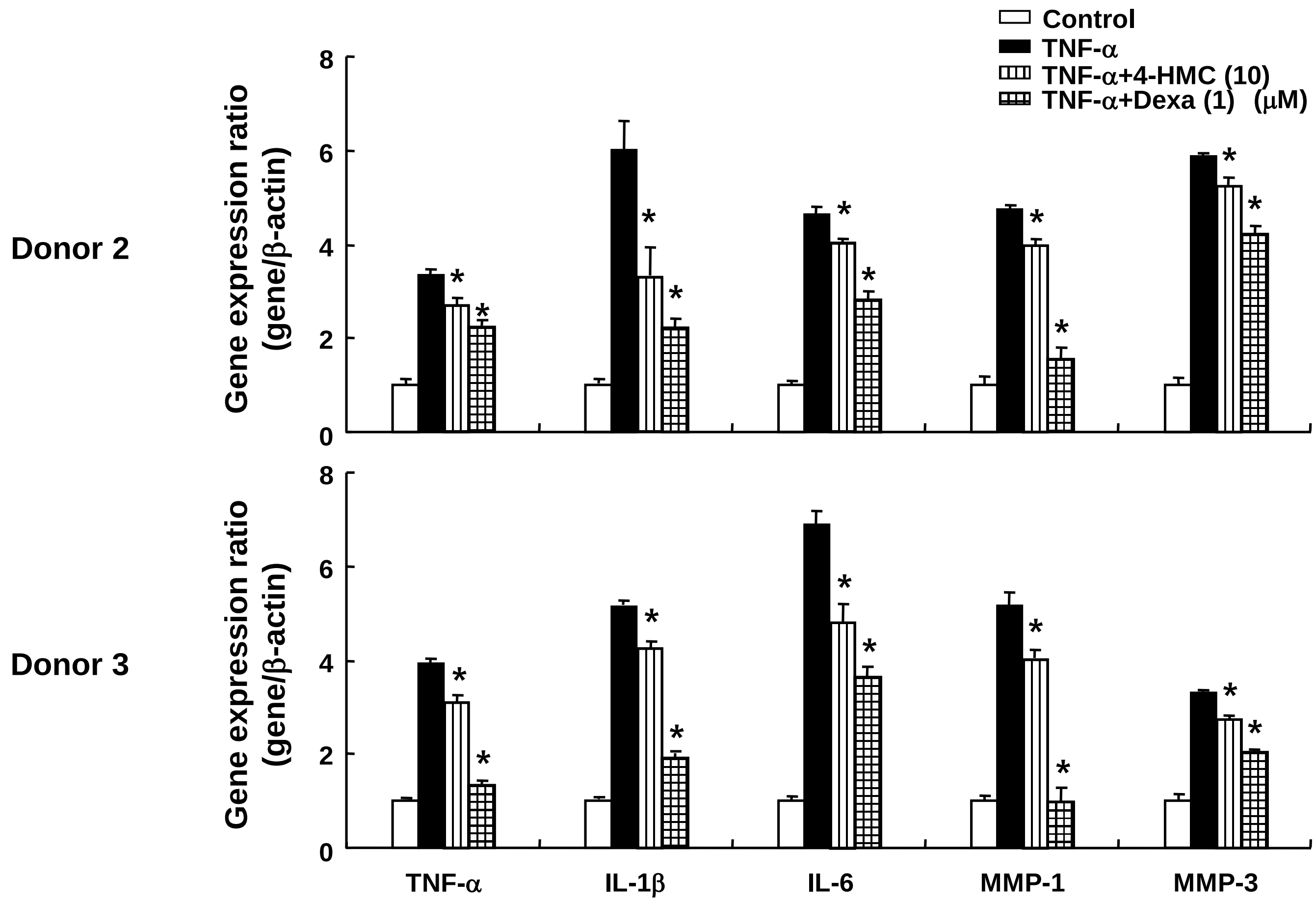

Supplementary Figure S7

Donor 2

Donor 3

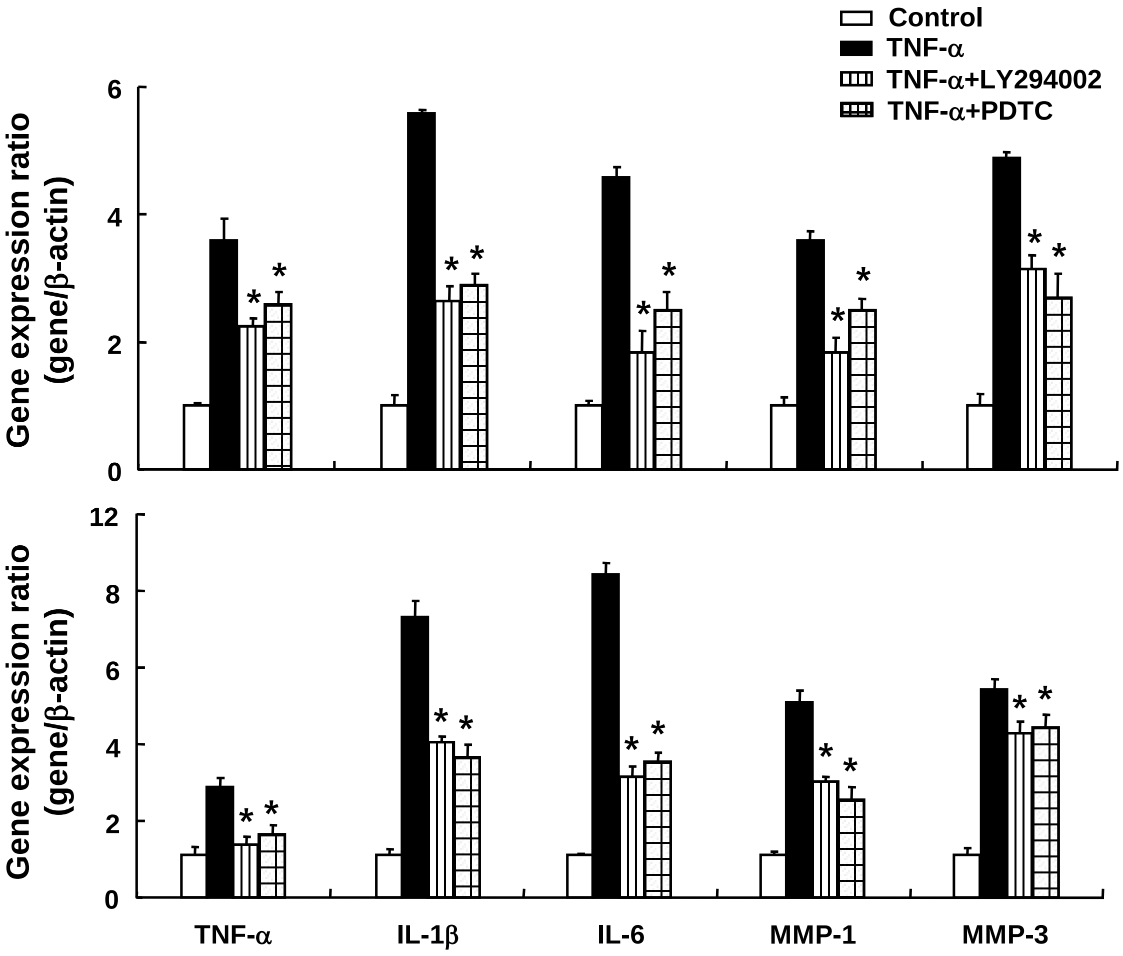

Supplementary Figure S8
